# Growth stimulation expressed gene 2 (ST2): Clinical research and application in the cardiovascular related diseases

**DOI:** 10.3389/fcvm.2022.1007450

**Published:** 2022-11-04

**Authors:** Jinchao Chen, Peng Xiao, Dan Song, Dewei Song, Zhi Chen, Hongmei Li

**Affiliations:** ^1^College of Materials and Chemistry, China Jiliang University, Hangzhou, China; ^2^National Institute of Metrology, Beijing, China; ^3^Key Laboratory of Chemical Metrology and Applications on Nutrition and Health for State Market Regulation, Beijing, China

**Keywords:** biomarker, sST2, cardiovascular disease diagnosis, clinical testing, traceability

## Abstract

As an interleukin (IL)-1 receptor family member, scientists found that when circulating soluble growth stimulation expressed gene 2 (sST2) is low, its ligand, IL-33, will bind to ST2L to exert protective effects on various types of cells. On the other hand, competitive binding of IL-33 occurs when sST2 concentrations are increased, followed by a reduction in the amount available for cell protection. Based on this mechanism, the usage of sST2 is to identify the population of high-risk patients with cardiovascular disease. In recent years, the role of serum sST2 in the occurrence, diagnosis, prognosis, and treatment of cardiovascular diseases has been gradually accepted by doctors. This manuscript systemically reviews the biological functions and applications of sST2 in disease diagnosis and treatment, especially for cardiovascular diseases. In clinical testing, since IL-33 can negatively impact sST2 measurement accuracy, the properties of current assay kits have been summarized and discussed to provide a clear view of the clinical chemistry results. Although sST2 is a promising biomarker, there are few quantitative approaches available for clinical testing. In this context, a mass spectrometry (MS)-based approach might be an option, as this is a powerful analytical tool to distinguish structurally related molecules in the matrix and decrease false-positive results in clinical testing. Moreover, approaches developed based on MS would be an ideal way to further study sST2 standardization.

## Introduction

### The background of growth stimulation expressed gene 2

#### Historical background of growth stimulation expressed gene 2

Growth stimulation expressed gene 2 (ST2) is part of the interleukin-1 (IL-1) receptor superfamily and was known as IL1RL-1 (1). As a new biological marker, ST2 plays an essential role in a variety of diseases since it can form a signaling pathway with IL-33, especially inflammation (1–3), immune diseases (4), and cardiovascular diseases (5). In recent years, the role of serum soluble ST2 (sST2) in the occurrence, diagnosis, prognosis, and treatment of cardiovascular diseases has been reported. This manuscript reviews the relevant functional roles of serum sST2 in cardiovascular diseases. Tominaga et al. induced mouse BALB/C-3T3 cells to produce a specific secretory protein by treating them with beta-interferon to study the G0/G1 phase transition mechanism of mouse cells in 1985 (6). ST2 was then first identified by two laboratories that studied growth-stimulated fibroblasts in 1989 (7). Tominaga et al. cloned this secretory protein by cDNA replication onto human chromosome 2Q12.1 and first confirmed that the protein had a high degree of sequence homology with members of the immunoglobulin superfamily, especially the extracellular portion of murine IL-1R (IL-1R1 and IL-18Rα) (8), and the gene product was named ST2 and is also known as DER4, IL1RL1, T1, and FIT-1. Weinberg et al. showed that the translation and expression of sST2 were abnormally upregulated in cardiomyocytes with mechanical damage when they analyzed the expression of 7,000 genes in cardiomyocytes suffering from mechanical strain in 2002. This result identified the role of ST2 in the cardiovascular system. Furthermore, serum concentrations of ST2 in mice were measured with a sandwich enzyme-linked immunosorbent assay (ELISA). According to the test results, a rapid increase in serum sST2 in mice within a few hours after myocardial infarction (MI) further demonstrated the physiological role of sST2 in the cardiovascular system (9). It was an active process that maintained a higher level of sST2 in the serum. In addition, Weinberg also found that sST2 is increased in the circulation of patients 1 day after MI. Subsequently, clinical studies further proved that the serum levels of sST2 in patients with cardiovascular disease was always maintained at a high level and was relevant to the risk of major adverse cardiovascular events (MACEs) and all-cause mortality (10). However, the biological function and mechanism of ST2 could not be further illustrated, and ST2 was considered an orphan receptor at that time until Schmitz et al. found that interleukin 33 (IL-33) could be used as a functional ligand of ST2 that researchers had a deeper understanding of the biological function of ST2 (11). Studies related to ST2 gradually increased.

ST2 has been a research focus in clinical cardiovascular disease since the American College of Cardiology Foundation/Heart Association (ACCF/AHA) formally listed sST2 as a biomarker of myocardial fibrosis in 2013. sST2 was used in the risk stratification of patients with heart failure (HF) in the 2013 ACCF/AHA guideline for heart failure management. It is clearly illustrated in the guideline that the recommendation of sST2 is class IIb and evidence level B in chronic heart failure and class IIb and evidence level A in acute heart failure. sST2 is not only the predictor of prognosis in patients with HF but is also additive to natriuretic peptide levels due to its prognostic value (12).

#### The structure and origin of growth stimulation expressed gene 2

**FIGURE 1:**
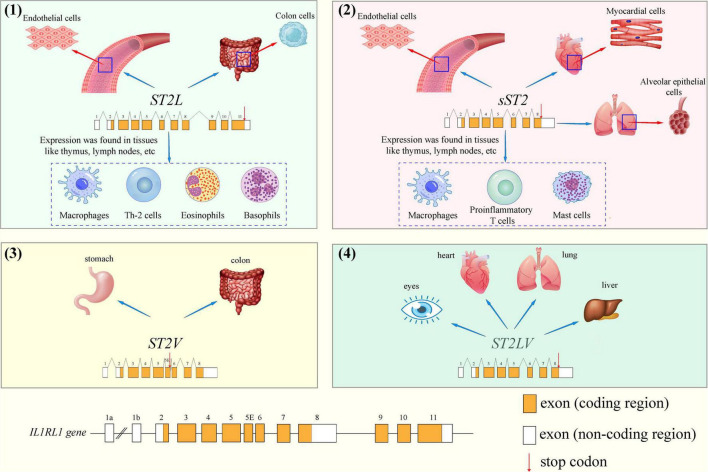
The different gene structures of stimulation expressed gene 2 (ST2) isoforms and cellular localization: **(1)** ST2L, **(2)** sST2, **(3)** ST2V and **(4)** ST2LV.

The gene that encodes ST2 is located on human chromosome 2q12 and belongs to the larger IL-1 gene cluster (GenBank accession number AC007248) (13, 14). As shown in Figure 1, the human ST2 gene encodes four isoforms by alternative promotor splicing: soluble ST2 (sST2 or IL1RL1-a), membrane-bound receptor ST2 (ST2L or IL1RL1-b), ST2V and ST2LV. Among the four isoforms, sST2 and ST2L are the most critical transcriptional products. As seen in Figure 1, different isoforms of ST2 have its own unique gene expression sequence (the red arrows indicate the stop codon). Variant 1 (ST2L consisting of 556 amino acids) represents the most extended transcript and encodes the full-length transmembrane isoform. ST2L is a type I membrane receptor containing three extracellular IgG-like domains, a transmembrane domain, and an intracellular Toll/IL-1R domain (15). ST2L is expressed in colon cells, endothelial cells, and various hematopoietic cells, such as basophils, CD4 + T lymphocytes, eosinophils, and macrophages (16, 17). Variant 2 (sST2, made up of 328 amino acids) is shorter than ST2L and has a unique C-terminus composed of nine and five amino acids in mice and human, respectively. As a soluble truncated form of ST2L, all transmembrane and Toll/IL-1 receptor (TIR) intercellular domains in ST2L are missing (18). Initially, researchers believed that sST2 was mainly secreted by myocardial cells, endothelial cells, and various immune cells such as proinflammatory T cells, macrophages, and mast cells, either constitutively or in response to stimulation (19). However, Pascual-Figal et al. showed intense sST2 immunostaining in the alveolar epithelium in 2018. Therefore, the lungs are probably a relevant source of sST2 in cardiovascular disease (20). It can be found that sST2 is expressed in many parts of the human body, and the specific secreted factors of sST2 need to be judged according to the disease type of patients with cardiovascular diseases. For example, in patients with myocardial injury, cardiomyocytes and endothelial cells are the significant secretors of sST2. Nevertheless, in hypertensive patients, immune cells associated with inflammatory processes probably are the main secretors of sST2. ST2V (composed of 259 amino acids) and ST2LV (composed of 211 amino acids) are splicing variants of ST2L due to the difference in exons: in ST2V, alternative splicing inserts a new exon, resulting in the acquisition of a new hydrophobic tail and the absence of the third immunoglobulin-like domain of ST2L (21). ST2V can be expressed in the stomach and intestines (22). ST2LV, which is found in the later stages of embryogenesis, lacks the transmembrane domains of ST2L and is secreted by many tissues, including the eyes, heart, lungs, and liver (23, 24).

#### The origin and biological function of interleukin-33

As essential functional ligands of ST2, IL-33 and ST2 both belong to the IL-1 family of cytokines. Onda H. et al. discovered a nucleoprotein encoded by the DVS27 gene when studying the molecular processes of continuous vasospasm in cerebral arteries (24, 25). This nucleoprotein was first identified in 2003 and was originally named nuclear factor from high endothelial venules (HEV) (25, 26). Later, Schmitz J. et al. identified the protein IL-33 (also known as C9ORF26, DVS27, NF-HEV, and IL-1F11) in 2005 (11, 26). The sequences for human and murine IL-33 have been mapped to chromosomes 9 (9p24.1) and 19 (19qc1) and encode proteins containing 270 (30 kDa) and 266 amino acids, respectively. Resembling IL-1α and HMGB-1, IL-33 acts as a traditional and intracellular nuclear factor with transcriptional regulatory properties, making it a dual function-cytokine (27). IL-33 acts as a proinflammatory or anti-inflammatory cytokine, depending on the type of activated cell, the stimulus, and the microenvironment (26). High mRNA expression of IL-33 in the brain, lung, stomach, skin, and spinal cord was revealed by expression analysis of human cDNA libraries. Reduced IL-33 expression was observed in the lymph tissue, kidney, spleen, heart, and pancreas. IL-33 is predominantly present in stromal cells, including endothelial cells, fibroblasts, and smooth muscle cells (5, 28).

IL-33 is usually located in the nucleus and is involved in cellular homeostasis by binding to chromatin and regulating RNA transcription (29). As shown in Table 1, Pro-IL-33 (IL-33_1–270_) protein contains the functional domain of the polypeptide terminal and is synthesized through direct processing by the Golgi apparatus and endoplasmic reticulum. However, the intracellular transport process of IL-33 is not clear. Chen et al. first discovered the molecular mechanism of IL-33 release from epithelial cells during type II immune response induced by allergen protease in 2022 (30). Studies have shown that protease exposure activates the assembly of stress granules (SGs) in epithelial cells, thereby promoting IL-33 nucleoplasmic transport. Protease stimulation induces cells to produce the N-terminal p40 fragment (p40 NT-Gsdmd) rapidly cleaved by Gasdermin D (Gsdmd), which can form pores in the cell membrane and directly promote the release of cytoplasmic IL-33 into the cell. These two mechanisms together effectively regulate the secretion of IL-33 in the nucleus, and provide a new therapeutic strategy for the intervention of IL-33-dependent airway allergic diseases. Subsequently, IL-33 secreted outside the cell does not need a processing enzyme responsible for maturation because IL-1 family ligand does not possess a typical signal peptide (31). IL-33 undergoes proteolytic cleavage by cathepsin G and elastase secreted from neutrophils to amplify IL-33 bioactivity when cells or tissues are damaged. Lefrancais Emma et al. cleaved full-length IL-33_1–270_ and found that the resulting peptides (IL-33_95–270_, IL-33_99–270_, and IL-33_109–270_) were 30-fold more potent than the pro-form for activating ILC2s *ex vivo* (32, 33). Moreover, these peptides with biological activity bound to ST2L receptor complexes in surrounding tissue cells through paracrine actions, mediating the immune response and subsequent reactions. During apoptosis, pro-IL-33 is secreted from the appropriate cell and contains a Caspase-3/7 cleavage site at aspartic acid, which can inactivate IL-33 and block the IL-33/ST2L signaling pathway, thereby avoiding the occurrence of autoimmune damage in surrounding tissues (34).

**TABLE 1:** The origins and biological functions of IL-33.

Variants	Origin	Biological function	References
IL-33_1–270_	As pro-IL-33	Released during cell necrosis and can activate the IL-33/ST2L signaling pathway	(31)
IL-33_178–270_	Cleaved by caspase 3/7	Cleaved and inactivated by caspases in cells undergoing apoptosis; unable to activate ST2 signaling	(34)
IL-33_95–270_	Processed by cathepsin G and elastase	The peptides with high biological activity can bind to the receptor ST2L	(32, 33)
IL-33_99–270_			
IL-33_109–270_			

The primary biological function of IL-33 is to bind to ST2L on the cell membrane to play a vital role in resisting many diseases. Next, the metabolic processes and mechanisms of the IL-33/ST2 signaling pathway will be introduced.

### The interleukin-33/growth stimulation expressed gene 2 signaling pathway

ST2L signaling pathway is activated by IL-33 to trigger pleiotropic immune responses in multiple ST2-expressing immune cells, such as B cells, type 2 helper T cells, and Basophils (35). In the cardiovascular system, IL-33 is mainly secreted by endothelial cells in the heart, binds to ST2L and has antihypertrophic and antifibrotic effects to protect the heart (36).

Based on the research in mice experimental models, Pascual-Figal et al. found that the IL-33/ST2 signaling pathway might protect the cardiomyocytes against hypertrophy, fibrosis, and cardiomyocyte apoptosis, thereby reducing cardiac dysfunction and improving patients’ survival rate (13, 36). Besides, sST2 will have a negative impact on the myocardium when IL-33 is separated. Seki et al. revealed that cardiomyocytes cultured with IL-33 could prevent hypoxia-induced apoptosis, and sST2 could partially inhibit this cardioprotective effect (37). The research by Miller also shows that the IL-33/ST2 signaling pathway seems to be involved in the mechanism of atherosclerosis as the administration of IL-33 in ApoE mice on a high-fat diet was related to the reduction of plaque size in the aortic sinus (38).

**FIGURE 2:**
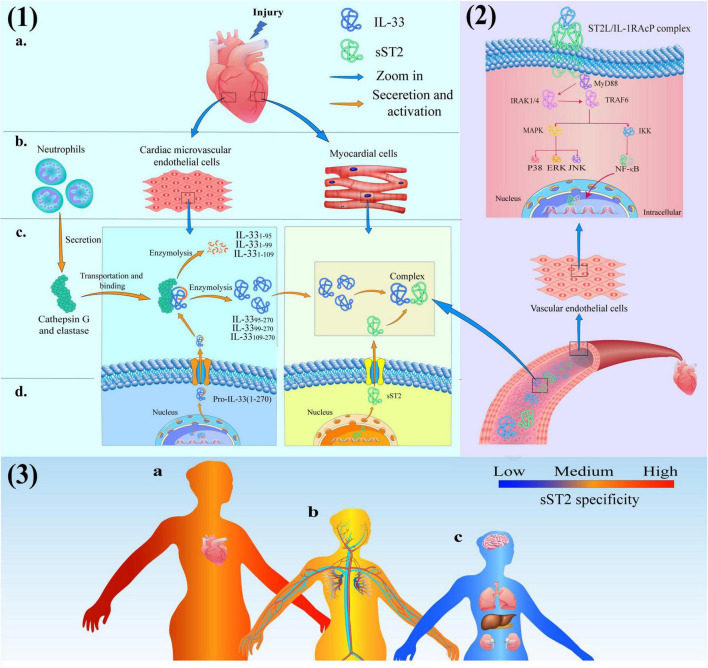
**(1)** Production and activation of IL-33 and soluble growth stimulation expressed gene 2 (sST2) protein in cardiac tissue consisted of (a) heart, (b) cells, (c) intercellular substance and (d) intracellular; **(2)** The IL-33/ST2 signaling pathway in Blood vessels; **(3)** The schematic diagram of sST2-related diseases: (a) Red-heart disease closely related to sST2 (high specificity); (b) Yellow-vascular diseases moderately correlated with sST2 (medium specificity) and (c) Bule-the diseases have limited association with sST2 (low specificity).

Firstly, the signaling mechanism of the IL-33/ST2 pathway in patients with myocardial injury is shown in Figure 2. For example, the protein expression of IL-33 and sST2 in endovascular epidermal cells and cardiomyocytes, respectively, is rapidly upregulated, and these proteins are secreted extracellularly, while cardiomyocytes or fibroblasts suffer mechanical damage due to cardiovascular disease (39). Afterward, secreted into the interstitial fluid can permeate through the capillary wall into the plasma and undergo proteolytic cleavage by cathepsin G and elastase to generate peptides with high biological activity. In the end, these peptides (IL-33_95–270_, IL-33_99–270_, and IL-33_109–270_) in the serum bind to the receptor complex composed of ST2L and IL-1RAcP due to its C-terminal IL-1-like cytokine domain. In response to the activation of ST2L/IL-1RAcP dimeric receptor complex, signal transduction results in the recruitment of myeloid differentiation primary response protein 88 (MyD88) by inducing the TIR domain of IL-1RAcP. Eventually, MyD88 binds to Toll like receptors (TLRs) on the ST2L/IL-1RACP Dimeric receptor complex (40).

IL-1 receptor-associated kinase 1 (IRAK 1) can maintain certain stability due to its combination with Toll-interacting protein (Tollip) when cells are resting. However, binding the C-terminal TIR domain of MyD88 to TLRs results in activation of the N-terminal DD domain of MyD88 and recruitment of IRAK family members. IL-1 receptor-associated kinase 4 (IRAK 4) first binds to the N-terminal DD domain of MyD88 at this moment and begins to act as a kinase to phosphorylate IRAK 1 (41). IRAK 1 reduces its affinity to Tollip due to phosphorylation and binds to MyD88 instead. At this time, the C-terminal TIR domain of MyD88 binds to the receptor, and the N-terminal DD domain recruits IRAK1, IRAK4 and TNF receptor-associated factor 6 (TRAF6) to the receptor. After activation, TRAK1 and TRAF6 were further phosphorylated and dissociated from MyD88. Subsequently, TRAF6 ubiquitinates its amino acid at position 63 under the catalysis of Ubc13/Uvel A. Ubiquitinated TRAF6 binds to and activates transforming growth factor β-activated kinase 1 (TAK 1) under the mediation of TAB-1 (TAK 1 binding protein-1) and TAB-2 (TAK 1 binding protein-2) (42). Activated TAK 1 phosphorylates mitogen-activated protein kinase (MAPK) and IκB kinase (IKK) complexes, causing two different pathways of signaling: the IKK pathway is activated to mobilize Ca^2+^ and subsequently activates the transcription factor (nuclear factor-κB, NF-κB). The MAPK pathway mediates the activation of extracellular signal-regulated kinase (ERK), p38, and Jun N-terminal kinase (JNK) and ultimately results in the production of cytokines, such as IL-1, IL-6, TNF-α (43, 44).

This signal results in gene transcription and the production of inflammatory cytokines/chemokines, which are secreted extracellularly and mount an adequate immune response to suppress inflammation, fibrosis, and ventricular remodeling to protect the heart. Furthermore, the expression of DNA in the nucleus also stimulates cell differentiation to exert unique biological effects. However, sST2 also binds to IL-33 as a decoy receptor, which can prevent the activation of the heterodimeric ST2L/IL-1RAcP dimeric receptor complex and block the protective effect of the IL-33/ST2 signaling pathway on heart tissue, since sST2 has the same extracellular domain as ST2L.

The inflammatory cytokines/chemokines released by the IL-33/ST2 signaling pathway are determined by the type of cells that express ST2L. Table 2 shows the role of the IL-33/ST2 signaling pathway in different immune cells and the production of inflammatory cytokines/chemokines (16, 45–53).

**TABLE 2:** Principal immune cells that response to IL-33.

Immune cell type	Function	References
B cells	Increase the number of B cells which can product circulating IL-10 Enhance the proliferation capacity of B1 cells and IgM, IL-5, and IL-13 production	(45)
Basophils	Promote the secretion of type 2 cytokines (such as IL-4 and IL-13)	(48)
Dendritic cells (DCs)	Promote the expression of MHC-II, CD40, CD80, CD86, OX40L, and CCR7 Prevent sST2 release Increase production of the allergy-associated cytokines and chemokines IL-4, IL-5, IL-13, CCL17, TNF-α, and IL-1β IL-33-induced DCs prime naive lymphocytes to produce IL-5 and IL-13	(47)
ILC2s	Promote the production of type 2 cytokine	(46)
Invariant natural killer T (iNKT) cells	Cause expansion and activation induce IFN-c in response to TCR engagement	(50)
M2-polarized macrophages	skew these macrophages toward an M2 phenotype Enhance activation by upregulating the lipopolysaccharide receptor components TLR4 and MD2, soluble CD14, and MyD88	(49)
Mast cells	Promote the maturation of mast cell, and induces the secretion of GM-CSF, IL-5, IL-13, CCL2, CXCL8, CCL17, and CCL22 accelerate the production of type 2 cytokines Promote the Th17 response during airway inflammation	(51, 52)
Natural killer (NK) cells	Increase IFN-c levels synergistically with IL-12	(50)
Regulatory T cells (Tregs)	Enhance protective effects/increases immunomodulatory functions increase Treg frequency and restrain IL-17 and IFN-γ productions	(53)
Th2 cells	Increase the expression of IL-4, IL-5, and IL-10 Inhibit the phagocytosis of macrophages	(16)
Eosinophils	Induce the production of degranulation, superoxide, and IL-8 Enhance Siglec-8 mediated apoptosis	(48)

## Application of soluble growth stimulation expressed gene 2 in cardiovascular diseases

The IL-33/ST2 signaling pathway plays an essential role in inflammation, immune diseases, and cardiovascular diseases; therefore, sST2 cannot be used as an independent diagnostic factor of cardiovascular disease. The primary function of sST2 is to act as a predictor to provide additional information for the treatment, prognosis, and risk assessment of patients with cardiovascular disease (54, 55). Regarding cardiovascular disease, the levels of serum sST2 are significantly correlated with the development of heart failure, coronary atherosclerotic heart disease (CAHD), type 2 diabetes mellitus (T2DM), and hypertension, as shown in Figure 2 (3). Meanwhile, the specific source of sST2, endpoint and proposed sST2 cutoff in various cardiovascular diseases are also listed in Table 3 (56–63).

**TABLE 3:** Studies evaluating the association of soluble growth stimulation expressed gene 2 (sST2) in various cardiovascular diseases.

Cardiovascular disease	The specific source of sST2	Proposed sST2 cutoff (ng/ml)	Endpoint	References
Acute heart failure	Cardiomyocytes and endothelial cells	35	All-cause death	(56)
		49	1-year all-cause death	(57)
Chronic heart failure	Cardiomyocytes and endothelial cells	28	All-cause and CV death, HF hospitalization	(58)
		35	Death or transplantation	(59)
Coronary artery heart disease	Cardiomyocytes	27.45	Sudden cardiac death	(60)
Diabetes mellitus, type 2	Immune cells	38.6	All-cause death	(61)
Hypertension	Immune cells and endothelial cells	28.6	1-year all-cause death	(62)
Myocarditis	Cardiomyocytes and immune cells	58.39	Optimal diagnosis of fulminant myocarditis	(63)

### Heart failure

Recent studies suggest that sST2 cannot be used as an independent diagnostic factor for heart failure (64) because it is involved in many disease processes, which reduces its specificity in diagnosing heart failure (65). Pascual-Figal D. et al. discovered that the serum concentration of sST2 was positively correlated with the severity of sudden cardiac death (SCD) in patients with stable heart failure in 2008. It was proposed to use sST2 to predict the mortality of HF patients and improve the prognostic accuracy of patients by combining it with NT-proBNP detection (66). Then, Pascual-Figal D. suggested that determining the sST2 concentration in serial serum samples could provide additional risk stratification in patients with decompensated heart failure. Repeated measurements of sST2 were suggested to help clinical decision-making of the attending physician in 2010 (67). Pascual-Figal D. et al. showed that continuous measurement of sST2 concentrations in HF patients could predict the progression of left ventricular remodeling and achieve prognostic accuracy in 2015, based on the fact that sST2 can be used to predict adverse outcomes in patients (68). Moreover, the reasonability of using an upper reference limit of 35 ng/ml was verified in the diagnosis of acute and chronic heart failure (69). Michele Emdin et al. assessed the independent prognostic value of sST2 in chronic heart failure during a median follow-up of 2.4-year (58). According to the study by Michele Emdin, a doubling of sST2 increased the risk of heart failure hospitalization, cardiovascular death, and all-cause death by 30, 25, and 26%, respectively. Meanwhile, Watson et al. evaluated the predictive value of longitudinal changes in B-type natriuretic peptide (BNP) concentrations and sST2 concentrations and reported that a statistically significant correlation exists between changes in sST2 and major adverse cardiovascular events (MACEs) in asymptomatic and event-free ambulatory patients with cardiovascular risk (70). Currently, the application of sST2 in heart failure is mainly through the combined detection of BNP to provide more predictive information. Frioes et al. evaluated the prognostic accuracy and complementarity of B-type natriuretic peptide (BNP) and sST2 levels in patients with acute heart failure at discharge (71). The research proved that the prognosis of patients with HFpEF is worse only when both biomarkers are higher than the median, and there is no difference between the elevation of only one biomarker and the low level of both biomarkers.

### Coronary artery heart disease

Elevated serum concentrations of sST2 are independent predictors of long-term all-cause mortality and provide supplementary prognostic information for patients with coronary heart disease. In a meta-analysis of 12,690 patients with acute coronary syndrome (ACS) in 2015, Gu L. noticed that ACS patients had higher baseline cycle sST2 concentrations and had a worse prognosis (72). In terms of clinical treatment, the primary percutaneous procedure for myocardial infarction has been improved significantly. Nevertheless, the no-reflow phenomenon restricts the therapeutic effect of this process. Somuncu Mu divided the patients based on the cutoff values and discovered that the probability of the no-reflow phenomenon in the high sST2 group was significantly higher than in the low sST2 group from the receiver operating characteristics analysis. This finding confirmed that sST2 was an independent predictor of the no-reflow phenomenon in STEMI patients (73). Furthermore, Gaggin H.K. et al. found that the serum concentration of sST2 was independently interconnected with invasively measured aortic pulse pressure in patients undergoing coronary angiography. This finding provides insight into the biological function of sST2 in aortic stiffening and implies that the serum concentration of sST2 may be a valuable biomarker of CAHD (74). Hack-Lyoung et al. investigated whether baseline serum soluble ST2 (sST2) levels can predict clinical outcomes in patients with stable coronary heart disease (CHD) (75). The research showed that the serum sST2 level was significantly higher in patients with MACEs than in those without (47.3 vs 30.6 ng/ml). Besides, Benjamin et al. evaluated the long-term prognostic value of sST2 in patients with stable CAD during the 9.8 years median follow-up time (76). In the cohort of CAD patients, the elevated concentration of serum sST2 was an independent predictor of long-term all-cause mortality and provided complementary prognostic information to natriuretic peptides. Liu et al. further confirmed this conclusion by using Meta-analysis (77). The result suggests that the higher concentration of baseline sST2 is relevant to the higher risk of MACEs, all-cause mortality, and HF in patients with CAD. Increased serum sST2 levels could significantly predict future MACEs in the ACS population (78).

### Diabetes mellitus, type 2

The cardiovascular complication of diabetes is the main reason for death in T2DM patients. Approximately 80% patients with T2DM will eventually die due to cardiovascular events. Therefore, assessing cardiovascular risk in patients with T2DM at an early stage will be helpful to control disease progression and reduce patient mortality. Bartunek et al. noted that patients with type 2 diabetes were more likely to be obese due to chronic adipose tissue inflammation. Thus, patients with T2DM are in a low-grade chronic inflammatory state (79). Vascular endothelial cells can rapidly and abundantly express sST2 in response to a large number of inflammatory factors, revealing the correlation between the serum concentrations of sST2 and the severity of diabetes. Evangelos et al. investigated the differences in the levels of sST2, BNP and hs-CRP between healthy controls and T2DM patients with and without left ventricular diastolic dysfunction (80). The research revealed that T2DM patients exhibit higher sST2 levels compared to healthy controls and there is also a significant relevance between sST2 levels and glycemic control. Lin YH observed that the levels of sST2 in T2DM patients were modestly but significantly elevated (26.1 ng/ml) compared with normal subjects (19.3 ng/ml) and persons with prediabetes (20.3 ng/ml), which demonstrated the statistically significant correlation between serum sST2 levels and risk of diabetes (81). Besides, Lin discovered the circulating sST2 levels were relevant to the levels of hepatic and renal function biomarkers and proposed the mechanism of hepatic function damage in patients is likely connected with abnormal immune responses under high glucose conditions because of the critical role of sST2/IL-33 pathway in inflammation. Li M et al. found that an elevated serum concentration of sST2 was significantly correlated with long-term MACEs and all-cause mortality in patients with and without T2DM in 2021. sST2 has substantial prognosis value for cardiovascular adverse events in T2DM patients, providing new cognition of the physiological function of sST2 (82). Lately, Fabian Hammer et al. also found that sST2 was a strong and independent predictor of fatal events in patients with T2DM by multivariate Cox proportional hazards analysis (83).

### Hypertension

At present, echocardiography has many limitations in evaluating the structural and functional changes in the left ventricle in hypertensive patients. Moreover, the use of biomarkers may better assess heart structure and function changes because inflammatory cells play an essential role in hypertension. Farcas et al. examined the relationship between the serum concentration of sST2 and diastolic dysfunction (DD) in patients with hypertension in 2017. Farcas found that serum sST2 levels were closely related to left ventricular remodeling and DD parameters, thus providing additional data for echocardiography as a valuable diagnostic biomarker of cardiac remodeling and diastolic functional changes in hypertensive patients (84). Subsequently, Wang et al. showed that the levels of serum sST2 increased progressively with the level in different essential hypertension (EH) groups (85). serum sST2 Levels of the LVH (left ventricular hypertrophy) group were higher than those of the NLVH (no left ventricular hypertrophy) group, and it is positively correlated with LVH-related indexes. Yin X. et al. proposed that sST2 was a risk factor for the occurrence of hypertension and could be used as a promising novel predictive biomarker for hypertension in 2019 (86). Indeed, as a marker of cardiac hypertrophy, the natriuretic peptide family can also assess heart structure and function changes well (87). Compared with natriuretic peptides, serum sST2 levels can respond to the development of hypertension and a variety of cardiovascular complications due to its low specificity. Therefore, serum sST2 levels can reflect the overall health status of patients with hypertension and thus better predict the prognosis of patients (62).

### Myocarditis

As a disease characterized by localized or diffuse inflammatory lesions in the myocardium, myocarditis is a significant cause of acute death and chronic heart failure, with an estimated worldwide incidence of 0.5–4.0%. While enhanced cardiac magnetic resonance imaging is an accurate technique for detecting areas of myocardial damage, there are many limitations to its use in emergencies.

Coronado et al. discovered that the serum concentrations of sST2 were elevated in men with myocarditis and associated with the NYHA class in male patients with myocarditis under 50 in 2019 (88). Research by Obradovic added further evidence to demonstrate the clinical relevance of sST2 and myocarditis-higher plasma levels of sST2 were related to lower values of the LV-EF in patients (89). Wang et al. measured the concentration of 39 cytokines in patients with fulminant myocarditis (FM) in 2022 to seek biomarkers that could promote the early diagnosis and treatment of FM (63). The results revealed that sST2 showed the most noteworthy dynamic changes from disease onset to resolution. These findings suggested that sST2 might be used as a potential biomarker for the rapid diagnosis of FM since FM is characterized by mechanical solid stretch stimulation and a severe inflammatory response.

## Measurement of serum soluble growth stimulation expressed gene 2 levels

### Available assays of soluble growth stimulation expressed gene 2 measurement

Serum sST2 can be measured by ELISA, amplified luminescent proximity homogeneous assay (AlphaLISA), and electrochemical immunosensing technology. These assays are all based on the principle of antigen-antibody binding, although the detection mode differs among these techniques. These detection methods will be described in detail.

#### Enzyme-linked immunosorbent assay

Currently, almost all clinical measurements of serum sST2 concentrations are completed by ELISA. The first ELISA for measuring sST2 in human serum/plasma was developed in 2000 (90). Table 4 shows information on commercially available assay kits for measuring sST2 in human serum/plasma (91).

**TABLE 4:** Information on commercially available assay kits for measuring soluble growth stimulation expressed gene 2 (sST2) in human serum/plasma (91).

Manufacturer	Assay/kit	Limit of detection (ng/ml)	Measurement range (ng/ml)	Intra-assay CV (%)	Interassay CV (%)
Critical diagnostics	Presage ST2 kit	1.3	Up to 200	<7	<9
MBL international	Human ST2 ELISA kit	0.032	Up to 20	<6	<6
R&D Systems	ST2/IL-1 R4 DuoSet ELISA or Quantikine ELISA	0.032	Up to 2.0	<6	<8
RayBiotech	Human IL-1 R4/ST2 ELISA kit	0.002	Up to 1.2	<10	<12

However, Mueller T compared the results of soluble ST2 measured by three different assay kits described above in 2012 and found that the results in plasma were different with the different kits (92). Therefore, the sST2 concentrations obtained by different kits were not consistent and could not be directly compared. The reason why this kind of difference occurs may be that the antibodies used in the different kits recognize different epitopes on the antigen, the purification process of the standard is different, and the reaction system is different. In addition, there is a lack of standard substances related to the sST2 protein worldwide, so it is difficult to standardize the measurement results of sST2 in each kit. The Presage^®^ ST2 assay kit is the only one approved by the U.S. Food and Drug Administration and is indicated to be used in conjunction with clinical evaluations to assess the prognosis of HF patients. Thus, the Presage^®^ ST2 assay kit with high accuracy (CV < 9%) and a comprehensive linear detection range is now the most widely used in the field of clinical research to precisely determine the level of sST2 in human serum (69).

#### AlphaLISA

**FIGURE 3:**
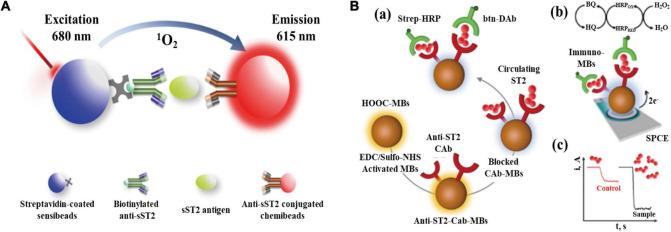
**(A)** Schematic representation of AlphaLISA for measuring soluble growth stimulation expressed gene 2 (sST2) through double-antibody sandwich immunocomplexes; **(B)** Preparation of the MB-based immunosensor for measuring sST2. (a) Steps in the sandwich immunoassay. (b) Electrochemical transducer and reactions involved in the amperometric readout. (c) Example of amperometric traces recorded for control and high ST2 samples (Have obtained the appropriate permissions form DMP and MDPI).

Although ELISA technology has become the only method for serum sST2 measurement in the field of clinical medicine with its advantages of high sensitivity, specificity, and diversified applications, it still has many disadvantages, such as requiring multiple washing steps, complex steps, a narrow dynamic range and high-affinity requirements for antibodies. Improving it on the premise of ensuring accuracy has become a focus of current research (93). As shown in Figure 3A, AlphaLISA uses microbeads as donors and receptors to detect biomolecules. Donor microbeads and recipient microbeads will become close to each other due to the specific recognition of antibodies when there is a measurand in the sample. The chemiluminescence reaction on the receptor microsphere will be amplified (94). Based on the principle of AlphaLISA, Gao SX and Li JP established and validated an original AlphaLISA for measuring serum sST2 (95). Briefly, a mixture of serum containing sST2 in patients, biotinylated antibodies, and suspensions of antibody-conjugated chemibeads were added to a 96-well microtiter plate. Then, the plate was incubated at 37°C under stirring conditions to compound sandwich-type immunocomplexes. Streptavidin-coated sensibeads were added and incubated under the condition of complete shading at 37°C with shaking. Finally, a chemiluminescence reaction was generated, and the signal intensity was regarded as the relative light units using the LICA reader. Furthermore, the assay exhibited high sensitivity with a limit of detection (LOD) of 0.176 ng/ml and a limit of quantification (LOQ) of 0.8 ng/ml, the intra-assay and interassay precision values were both less than 15%.

However, since the AlphaLISA technique is viewed as a wash-free method, it may be more susceptible to disturbed by abnormal serum samples due to jaundice, hemolysis, and hyperlipemic blood. Additionally, biotin causes interference in many streptavidin-biotin-based immunoassays, leading to false and biased results in sandwich immunoassays.

#### Electrochemical immunosensing technology

According to the 2010 International Union of Pure and Applied Chemistry (IUPAC) classification criteria for biosensors (96), electrochemical immunosensing technology is a self-contained integrated technology based on the antigen-antibody reaction that can be used to perform specific quantitative or semiquantitative analysis. Antigen-antibody binding is a molecular recognition element and makes direct contact with the electrochemical sensing element. Finally, the sensing element can convert the chemical concentration signal into the corresponding electrical signal.

Based on the principle of electrochemical immunosensing technology, Demirbakan B and Sezginturk MK fabricated an electrochemical immunosensor with high sensitivity based on a fullerene C_60_-modified disposable graphite paper (GP) electrode for measuring sST2 concentrations in human serum. The advantages of this technology are its reusability, high sensitivity, and excellent reproducibility (97). Electrochemical impedance spectroscopy, cyclic voltammetry, and the single frequency impedance make up the sST2 electrochemical immunosensor, which was used to examine the specific interaction between sST2 antigens and anti-sST2, and the serum concentration of sST2 was obtained by converting to electrical signals. This sST2 electrochemical immunosensor had superior detection accuracy, repeatability, and a wide detection range. Moreover, the proposed immunosensor had a low LOD and LOQ values of 0.124 and 0.414 fg/ml, respectively.

Torrente-Rodriguez R.M. et al. reported a novel magnetic bead (MB)-associated amperometric immunosensor for measuring sST2, which could measure the serum concentration of sST2 in only 45 min once the immunoconjugates were formed (98). The structure and detection mechanism of the immunosensor are shown in Figure 3B. A sandwich immunoassay and disposable screen-printed carbon electrodes were used in this method. Magnetic immunoconjugates built on the surface of carboxylic acid-microsized magnetic particles (HOOC-MBs) were used to selectively capture sST2. The biotinylated secondary antibody was further conjugated with a streptavidin peroxidase conjugate (Strep-HRP) and was used to sandwich the target protein. This immune platform exhibits excellent measurement accuracy and a low LOD (39.6 pg/ml) for sST2.

### The specificity of soluble growth stimulation expressed gene 2 assays

**FIGURE 4:**
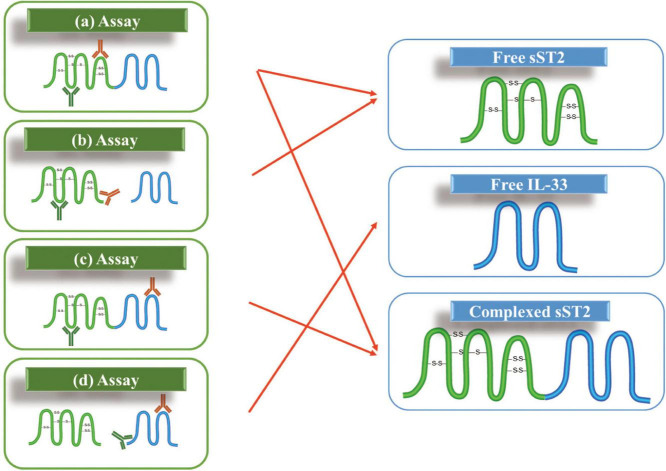
Possible assay formats for measuring soluble growth stimulation expressed gene 2 (sST2) and IL-33 in the circulation: (a) two epitopes involved in sST2; (b) one antibody against an epitope on sST2 while another one against the binding site of IL-33; (c) one antibody against an epitope on sST2 while another one against an epitope on IL-33; and (d) two epitopes involved in IL-33.

These assays are all based on the principle of antigen-antibody binding. The specificity of sST2 assays needs to be evaluated. As shown in Figure 4, sST2 and IL-33 is measurable in different forms in circulation. Theoretically, the three analytes: free sST2, complex of sST2 and IL-33, and free IL-33 should be present in the circulation. Therefore, there are many different combinations due to the differences in antibodies recognizing epitopes. As seen in Figure 4, there are four possible detection combinations: free sST2, sST2 complexed, both free sST2 and sST2 complexed, and free IL-33. However, the situation is even more complex because different isoforms of IL-33 may be present in the circulation (IL-33_1–270_ can be processed by elastase and cathepsin G to produce IL-33_95–270_, IL-33_99–270_ and IL-33_109–270_) as well as the measurement of IL-33 also depends on the epitopes recognized by the respective antibodies (these issues are not shown in Figure 4).

This challenge has not been reasonably addressed since it was raised by Thomas Mueller in 2015 (18). Thomas Mueller could not determine the type of sST2 detected in the serum to date; he presumed that the sum of free sST2 and complex of sST2 and IL-33 would be measured with the ELISA kits described. Later, Alberto Aimo showed this difficulty again in his review in 2019 (99). However, the authors believed the conjecture of Thomas Mueller and regarded the measurement result as the sum of free sST2 and compound sST2.

However, improving the measurement specificity is an urgent requirement for sST2 in clinical treatment. sST2 is a new biological marker, and the concentration in serum is correlated with the risk assessment of the diagnosis, treatment, and prognosis of cardiovascular patients. Only by defining the measurement specificity of serum sST2, doctors can determine the progression of cardiovascular disease in patients.

## Summary and prospects

Based on many fundamental studies in recent years, the role of sST2 in pathological conditions has become clear. However, the difficulties regarding accurate analysis of biological matrix sST2 mainly involve two aspects: measurement specificity and a value traceability system. Since the clinical diagnostic reagent might recognize IL-33 and the sST2 complex or free sST2, the testing results likely represent the total value of circulating molecules. As the biological activities of free and bound sST2 are quite different, the undefined targets might bring uncertainty to the testing values. Therefore, chemical analytical approaches, such as liquid chromatography or capillary electrophoresis tandem mass spectrometry (LCMS or CEMS), might be a powerful tool for native biomarker analysis.

Furthermore, when a laboratory-developed test (LDT) developed a novel method for matrix sST2, an issue related to the value assignment appeared. According to the latest public information, both the primary reference material (RM) and measurement procedure (RMP) for sST2 are currently unavailable. A lack of higher-level standards will result in a gap between the international system of units (SI units) and the manufacturer’s product. Then, the clinical testing results can only be traced back to the manufacturer’s selected measurement procedure or calibrator selected by the manufacturer, resulting in incomparability of clinical testing results from different laboratories or different assays. However, this does not mean that the RM and RMP are unique methods to clinical standardization, and coordinated research is also a viable way to mutual recognition of clinical test results. If so, trueness verification plan and proficiency test would be the foundational work that needs to be organized.

Researching and developing sST2 immunoassays based on antibodies is vital for clinical laboratory medicine, which solves the problem of creating something from scratch. The separating tool tandem MS is an efficient analytical method for assay specificity verification, as the different forms of sST2 would be conveniently separated by LC or CE technology. A key study in RM research is to develop an SI-traceable quantitative method. For this purpose, isotopic dilution and MS quantification of amino acids or signature peptides that were hydrolyzed or enzymatically digested from sST2 will help metrological researchers develop the certified RM. Additionally, the use of stable isotopic-labeled intact proteins as intermediates during RM development is the most preferred approach since no additional chemical reaction should be performed, which will help reduce the uncertainty of the final result. The most suitable RM from the upper reference laboratory to the end user is actually serum or plasma sST2, instead of the pure sST2 RM. Biological matrix cleanup and low-abundance target enrichment are still challenges in proteomics. Fortunately, since sST2 is expressed in human serum at the sub-milligram per liter level, several commercial extraction products can be used. The gaps in the value traceability chain may be bridged when the primary certified RM or RMP is developed.

## Author contributions

JC: investigation and writing – original draft. PX: conceptualization, and writing – review and editing. DeS and DaS: resources. ZC: validation. HL: resources and supervision. All authors contributed to the article and approved the submitted version.

## Abbreviations

ACS: acute coronary syndrome
CAHD: coronary atherosclerotic heart disease
DD: diastolic dysfunction
ELISA: enzyme-linked immunosorbent assay
FM: fulminant myocarditis
HEV: high colorectal venules
HF: heart failure
IL-1: interleukin-1
IL-33: interleukin-33
LOD: limit of detection
LOQ: limit of quantification
MACEs: major adverse cardiovascular events
RM: reference material
RMP: reference measurement procedure
ST2: growth stimulation expressed gene 2
T2DM: type 2 diabetes mellitus.
